# Acute Exercise-Induced Compartment Syndrome Following High-Load Resistance Training in a Powerlifter: A Case Report

**DOI:** 10.7759/cureus.105008

**Published:** 2026-03-10

**Authors:** Bradley Moushon, Elias Kondilis, Almir Music, Frederick Tiesenga

**Affiliations:** 1 General Surgery, Community First Medical Center, Chicago, USA; 2 General Surgery, West Suburban Medical Center, Chicago, USA

**Keywords:** acute care surgery and trauma, anabolic steroid abuse, emergency fasciotomy, emergency medicine and trauma, four compartment fasciotomy, lower extremity compartment syndrome, lower extremity trauma, non-traumatic compartment syndrome, non-traumatic rhabdomyolysis, sports-related

## Abstract

Acute compartment syndrome (ACS) is a surgical emergency most commonly associated with trauma. A less common variant, acute exercise-induced compartment syndrome (AEICS), occurs without direct injury and is rarely seen in strength athletes. We report a case of a 50-year-old male powerlifter who developed AEICS of the right lower leg following an 815-lb squat. He presented with delayed onset right calf pain, weakness, and creatine kinase (CK) elevation exceeding 93,000 IU/L. Despite an emergent four-compartment fasciotomy, he sustained a persistent right foot drop. This case highlights the diagnostic challenge of AEICS in nontraumatic settings, the potential role of anabolic steroids as a predisposing factor, and the importance of timely recognition and decompression to prevent permanent neurologic deficits.

## Introduction

Acute compartment syndrome (ACS) arises when elevated pressure within a closed fascial compartment compromises tissue perfusion, leading to ischemia and potential necrosis [1]. ACS most commonly follows trauma or vascular injury and requires urgent surgical decompression to prevent irreversible damage [2-6]. In contrast, chronic exertional compartment syndrome (CECS) develops gradually during repetitive activity and is typically managed nonoperatively [2].

Acute exercise-induced compartment syndrome (AEICS) represents a rare and distinct clinical entity within the spectrum of compartment syndromes. In contrast to traumatic ACS, which has an estimated incidence of approximately 3-4 cases per 100,000 persons annually, AEICS lacks a defined population-based incidence and has been described predominantly through isolated case reports and small case series [3,7,8]. The literature suggests that exercise-related cases comprise only a small minority of reported ACS presentations, contributing to frequent diagnostic delay and underrecognition [7,9]. AEICS most commonly occurs in young, otherwise healthy individuals following intense or unaccustomed physical exertion and may develop in the absence of trauma, anticoagulation, or underlying coagulopathy [7,10].

Extreme exertion can lead to rapid intramuscular swelling and fluid shifts within noncompliant fascial compartments, impairing perfusion and precipitating ischemia [5,6]. Proposed predisposing factors include muscular hypertrophy, tight fascial compartments, and anabolic-androgenic steroid use, although supporting data remain limited [5,6]. The objective of this report is to increase clinician awareness of AEICS, highlight distinguishing clinical features, and discuss potential contributing risk factors, including anabolic steroid use.

## Case presentation

A 50-year-old male powerlifter presented to the emergency department after a witnessed tonic-clonic seizure and fall. The day prior, he had performed an 815-lb maximal squat as part of his training regimen. His past medical history included seizure disorder, post-traumatic stress disorder, traumatic brain injury, obstructive sleep apnea, asthma, obesity, and a stable 3 mm cerebral aneurysm. He reported recent use of anabolic steroids, including testosterone and oxymetholone (Anadrol).

On presentation, he was alert but disoriented (AOx2) and subsequently intubated for airway protection. Initial labs showed a creatine kinase (CK) level of 48,050 IU/L, which peaked at 93,940 IU/L within nine hours. Creatinine was 2.69 mg/dL, aspartate aminotransferase (AST) was 681 IU/L, alanine aminotransferase (ALT) was 172 IU/L, and lactic acid was 7.2 mmol/L. These findings were consistent with rhabdomyolysis and acute kidney injury (AKI).

During his ICU course, he experienced multiple seizures requiring a continuous midazolam infusion. Five days after admission and discontinuation of midazolam, he developed progressive right calf pain, new paresthesia between the first and second toes, and inability to dorsiflex the ankle and toes. Examination revealed severe pain with passive dorsiflexion, weakness in both dorsiflexion and plantarflexion, and intact peripheral pulses.

**Figure 1:**
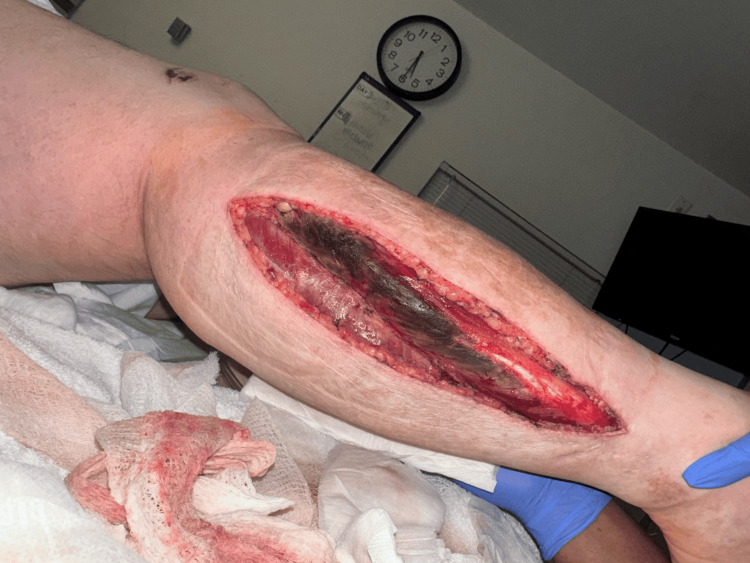
Postoperative day (POD) 2 fasciotomy Lateral incision of the right lower leg showing the lateral compartment with muscle necrosis. The photograph was taken during a dressing change.

**Figure 2:**
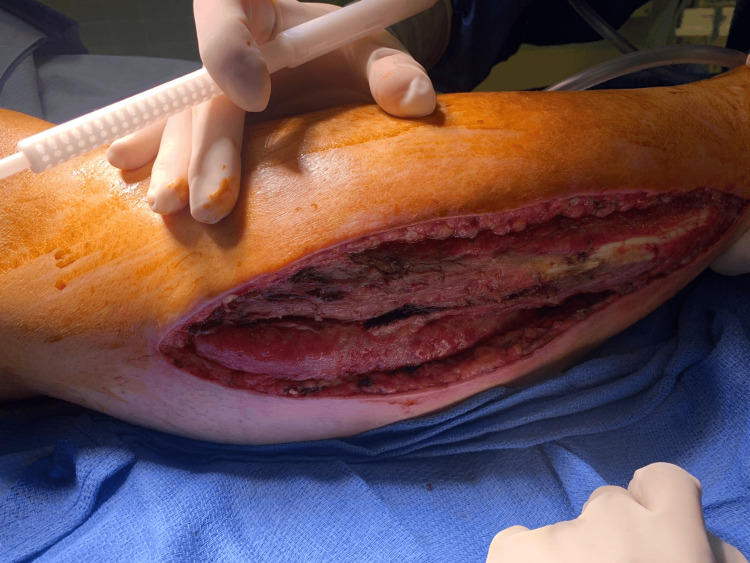
Postoperative day (POD) 6 debridement Surgical debridement of the right lower leg to remove necrotic muscle.

Given these findings, AEICS was suspected, and general surgery was consulted. Compartment pressure measurements were not obtained as the hospital lacked the appropriate instrumentation, and the diagnosis was made clinically. Emergent four-compartment fasciotomy of the right lower leg was performed. A lateral incision was made through the skin and subcutaneous tissue to expose the fascia. Inspection of the lateral fasciotomy site demonstrated effective decompression of the lateral compartment, though the muscle was bulging and showed signs of necrosis (Figure 1). The anterior compartment demonstrated marked bulging and pale muscle without contractile response, confirming nonviable tissue. The lateral compartment was released with minimal bulging and viable musculature (Figures 1-2).

**Figure 3:**
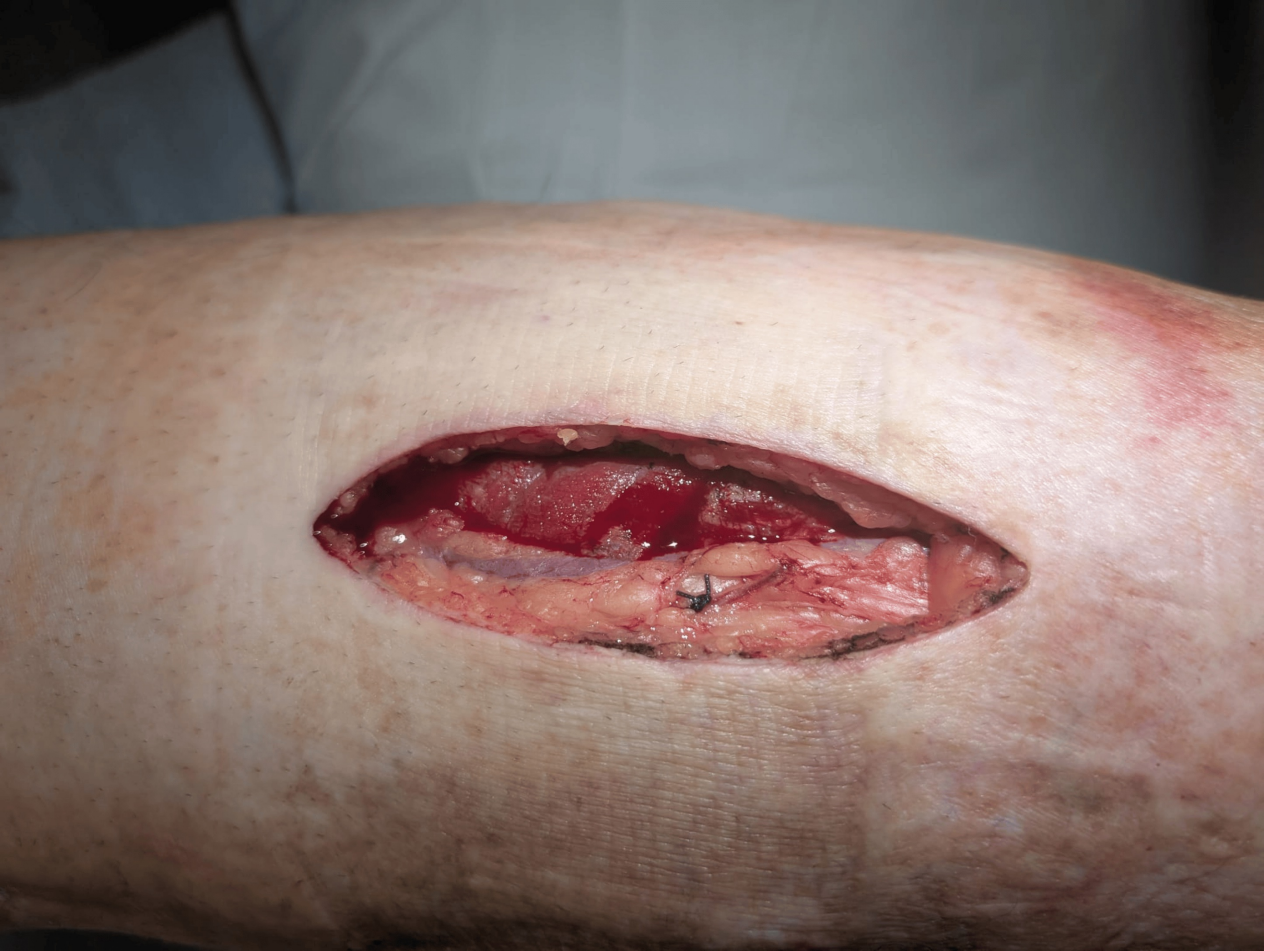
Postoperative day (POD) 2 anterior compartment fasciotomy Anterior compartment fasciotomy site showing decompression with exposed musculature and expected early postoperative changes.

A medial incision was subsequently made to access the superficial and deep posterior compartments. The superficial posterior compartment appeared healthy and well perfused (Figure 3). The deep posterior musculature appeared viable without necrosis. Hemostasis was achieved using electrocautery. Wet-to-dry dressings were applied, with plans for serial debridement of necrotic tissue in the anterior compartment (Figure 1).

**Figure 4:**
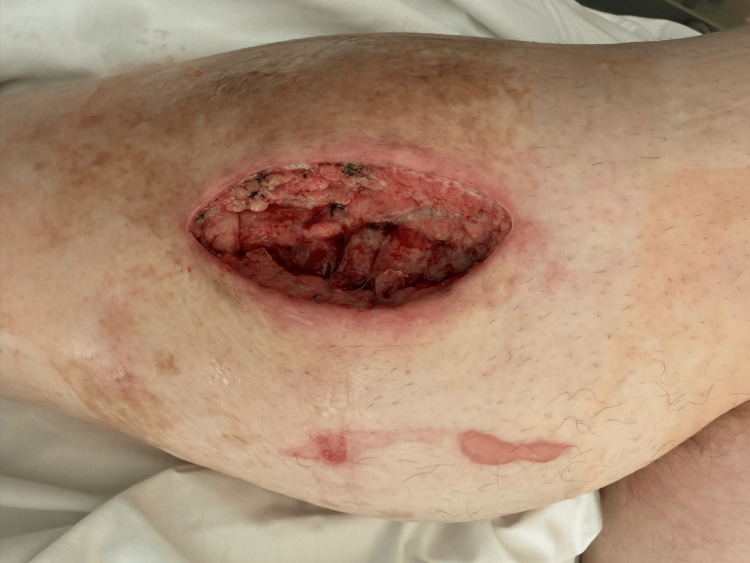
Postoperative day (POD) 5 Anterior compartment showing an open wound with tissue and exposed musculature. The wound edges appear viable without evidence of infection, consistent with expected postoperative healing.

**Figure 5:**
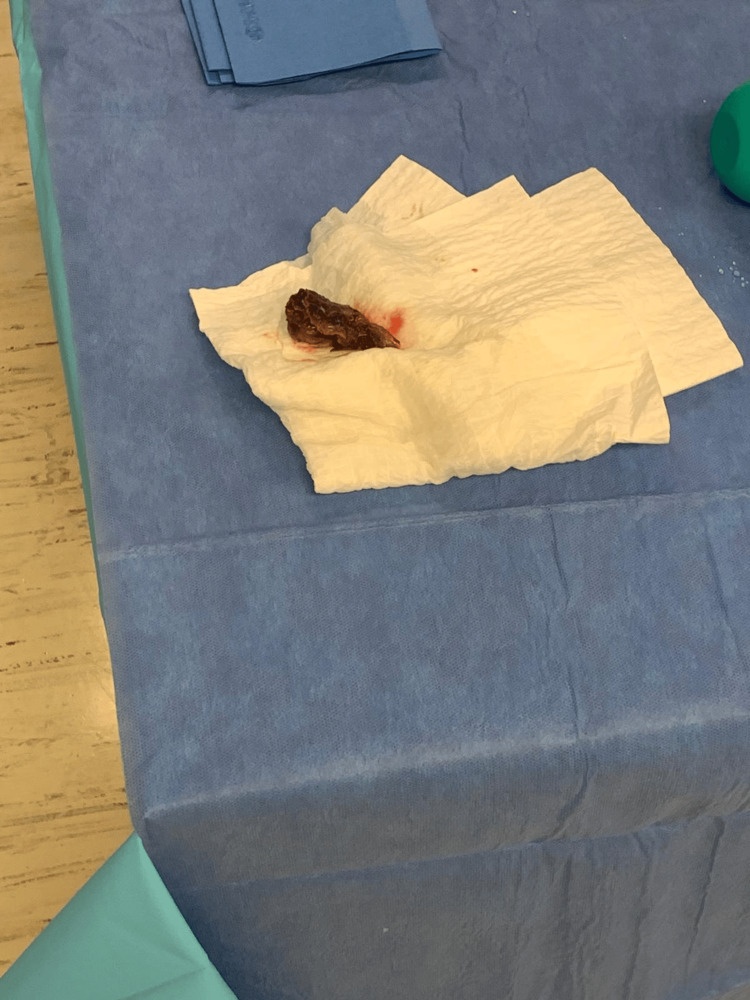
Necrotic tissue specimen Surgical pathology specimen of necrotic muscle excised from the lateral compartment during debridement.

**Figure 6:**
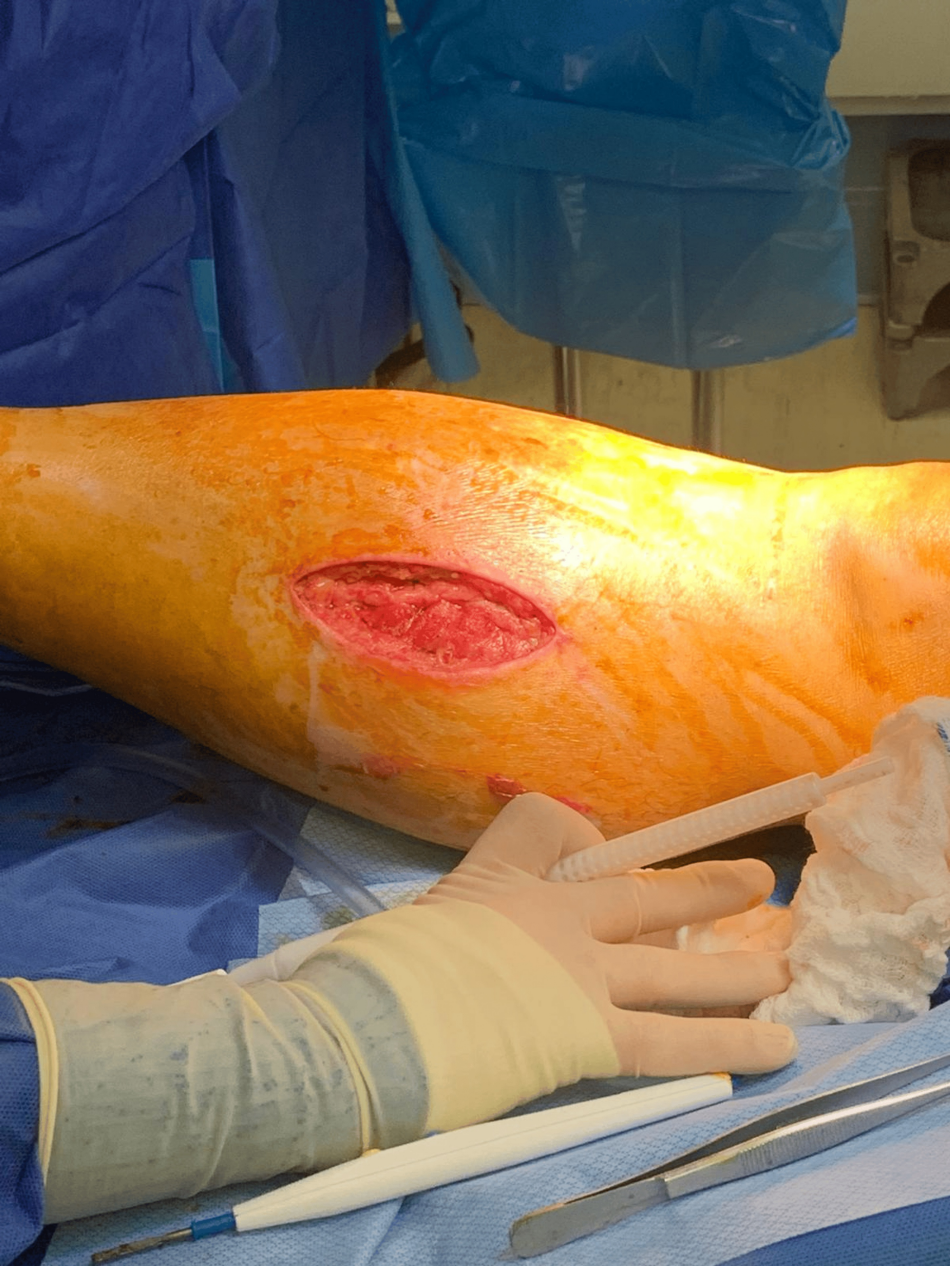
Postoperative day (POD) 6 Anterior compartment demonstrating a clean wound bed with exposed viable tissue.

Postoperatively, the patient continued to experience right foot drop. He underwent serial wound washouts, and by day 5, the fasciotomy wound remained open with healthy-appearing granulation tissue and no evidence of infection or further necrosis (Figure 4). On postoperative day 6, debridement of necrotic tissue from the lateral compartment (10 × 30 cm) was performed (Figures 5-6). Pathology was unremarkable, and he was discharged with a wound vacuum-assisted closure device (Genadyne Wound Vac System Model XLR8+; Genadyne Biotechnologies, Inc., Hicksville, USA) in place. At discharge, the wound showed healthy granulation tissue with no evidence of infection.

## Discussion

Acute and chronic compartment syndromes differ in both etiology and management. ACS most commonly results from trauma or vascular injury and requires emergent surgical decompression to prevent irreversible ischemia and tissue necrosis. In contrast, CECS presents with exertional pain that resolves with rest and is typically managed conservatively [1,2]. AEICS is a rare and often underrecognized entity that occurs without trauma and is driven by rapid intramuscular swelling and fluid shifts within noncompliant compartments, leading to impaired perfusion and ischemia [1,7,11].

AEICS is usually precipitated by extreme exertion, particularly in individuals with predisposing factors such as anabolic steroid use, congenital fascial tightness, or hypertrophic musculature [3-5]. The present case is notable for the exceptionally high exertional load (815-lb squat), which exceeds those reported in most prior cases. Concomitant anabolic steroid use likely contributed to increased muscle bulk and fluid retention, predisposing the patient to elevated compartment pressures and a severe presentation [3,5,6].

A key feature of this case was the diagnostic delay. Unlike most reported AEICS cases, where patients present promptly with exertional pain and swelling, recognition was delayed due to recurrent seizures and a medically induced coma. This delay likely contributed to irreversible neurologic injury, manifesting as persistent right foot drop, a complication less frequently reported in prior cases where timely fasciotomy often results in full recovery [3-6], as seen in Table 1.

**Table 1:** Lower leg compartments, clinical findings, frequency, outcomes, and complications in AEICS *Frequency reflects relative compartment involvement reported in published reviews and AEICS case reports rather than precise incidence rates [3,7,9] AEICS: acute exercise-induced compartment syndrome

Compartment	Key Clinical Findings	Approximate Frequency*	Typical Outcomes	Complications
Anterior	Severe shin pain, swelling, and paresthesia over the dorsum of the foot [1,2,6,11,12]	Most common [1,2,6,12]	Early fasciotomy restores function; delayed diagnosis may cause ankle dorsiflexion weakness or sensory deficits [1,6,12]	Persistent ankle dorsiflexion weakness, sensory loss [1,6,12]
Lateral	Lateral leg pain, swelling, and weakness of foot eversion [1,2,6,12]	Less common [1,2,6,12]	Early intervention usually prevents long-term deficits [1,6,12]	Foot eversion weakness if delayed [1,6,12]
Deep posterior	Calf tightness, pain on passive toe dorsiflexion, and plantar sensory changes [1,2,6,11,12]	Common [1,2,6,12]	Delayed decompression may result in persistent plantarflexion weakness or claw toes [1,6,12]	Plantarflexion weakness, claw toes, chronic pain [1,6,12]
Superficial posterior	Calf tenderness and tightness; milder pain [1,2,6,12]	Rare [1,2,6,12]	Outcomes favorable with early fasciotomy [1,6,12]	Rare; typically minimal [1,6,12]

The pathophysiology of AEICS involves exertion-related swelling and fluid shifts that acutely increase intramuscular pressure. When compartment pressures exceed 30 mmHg, or the delta pressure (diastolic minus compartment pressure) falls below 30 mmHg, tissue perfusion becomes critically impaired [7,11]. Although direct compartment pressure measurement remains the diagnostic gold standard, the diagnosis is often clinical and based on pain out of proportion to examination, pain with passive stretch, paresthesia, and weakness [12,13].

Anabolic steroid use likely contributed to both AEICS and exertional rhabdomyolysis in this patient. The markedly elevated CK level (>93,000 IU/L) and associated AKI were consistent with exertional rhabdomyolysis, which can further exacerbate compartment pressures through inflammatory edema and myocyte breakdown [14]. Although generalized tonic-clonic seizures may cause CK elevation, reported levels are typically lower. The magnitude, rapid rise, limb localization, and temporal relationship of CK elevation preceding seizure activity in this case support an exertional rhabdomyolysis and compartment syndrome mechanism rather than a postictal process [15].

Previously reported AEICS cases typically involve younger patients who present with exertional pain and swelling and recover fully following a timely fasciotomy [3-6]. The current case differs with respect to patient age, exertional load, pharmacologic exposure, and clinical outcome. Table 2 compares this case with a few previously published reports [3,7,9].

**Table 2:** Comparative summary of reported AEICS cases AEICS: acute exercise-induced compartment syndrome; CK: creatine kinase; M: male

Source	Patient/Age	Trigger/Activity	Compartment(s) Involved	CK (IU/L)	Outcome
Bhalla and Dick-Perez [3]	22 M football player	Strenuous weight lifting	Anterior, lateral	>200,000	Full recovery
Eldessouky et al. [6]	36 M	Intense upper-body exercise involving pull-ups	Upper extremity compartments	156,000	Full recovery
Dewangan et al. [7]	54 M	Vigorous heavy-weight lifting exercise while intoxicated	Anterior	37,599	Full recovery
Present case	50 M powerlifter	815-lb squat	All four compartments (anterior, lateral, posterior, superficial/deep)	93,940	Persistent right foot drop

Timely fasciotomy remains critical, as delays beyond six hours are associated with muscle necrosis, nerve injury, and long-term functional deficits, including limb loss [16]. Despite surgical intervention, this patient developed persistent right foot drop, underscoring the consequences of delayed recognition in atypical, nontraumatic presentations.

Limitations of this report include the absence of compartment pressure measurements, potential confounding from exertional rhabdomyolysis and AKI, and the inherent limitations of a single-case design. Nevertheless, this case highlights extreme exertional load and anabolic steroid use as potential risk factors and reinforces the importance of maintaining a high index of suspicion for AEICS in patients presenting with severe post-exertional leg pain, swelling, or neurologic deficits, even in the absence of trauma.

## Conclusions

AEICS should be considered in patients presenting with severe post-exertional leg pain, progressive swelling, and neurologic deficits, even in the absence of trauma. Although ACS is most commonly associated with fractures or high-energy injuries, this case demonstrates that strenuous physical exertion alone can be a sufficient precipitating factor. The lack of an external traumatic event may delay recognition, increasing the risk of serious morbidity. Thus, clinicians should maintain a high index of suspicion when pain is disproportionate to clinical findings or persists despite conservative measures. Prompt diagnosis and timely surgical fasciotomy remain the cornerstones of management, as delays markedly increase the risk of irreversible neuromuscular damage and functional impairment. This case also highlights the potential contribution of anabolic steroid use to AEICS through muscle hypertrophy, edema, and vascular compromise, as well as the possibility of delayed symptom onset following exertion, which broadens the spectrum of recognized presentations.

Importantly, this report underscores the need for increased awareness of AEICS among emergency physicians, sports medicine specialists, and surgeons. Early recognition, decisive intervention, and interdisciplinary collaboration are critical for preventing permanent morbidity and preserving limb function, particularly as intense weightlifting and performance-enhancing drug use become more prevalent.
